# Highly divergent patterns of genetic diversity and evolution in proviral quasispecies from HIV controllers

**DOI:** 10.1186/s12977-017-0354-5

**Published:** 2017-05-02

**Authors:** Suwellen S. D. de Azevedo, Diogo Gama Caetano, Fernanda H. Côrtes, Sylvia L. M. Teixeira, Karina dos Santos Silva, Brenda Hoagland, Beatriz Grinsztejn, Valdilea G. Veloso, Mariza G. Morgado, Gonzalo Bello

**Affiliations:** 1 Laboratório de AIDS & Imunologia Molecular, Instituto Oswaldo Cruz - FIOCRUZ, Av. Brasil 4365, Rio de Janeiro, RJ 21045-900, Brazil; 2 Instituto Nacional de Infectologia Evandro Chagas - FIOCRUZ, Av. Brasil 4365, Rio de Janeiro, RJ 21045-900, Brazil

**Keywords:** HIV-1, Elite controllers, Viremic controllers, Reservoir, Diversity, Evolution, Reseeding

## Abstract

**Background:**

Ongoing intra-host HIV-1 evolution has been shown in individuals that naturally suppress the viremia to low levels (HIV controllers) by the analysis of the RNA in plasma compartment. Detection of evolution at the DNA proviral compartment in HIV controllers, however, has been more challenging and the precise correlation between the systemic viral suppression level and rate of reservoir’s reseeding in those individuals is not fully understood. In this sense, we examined the proviral DNA quasispecies by single genome amplification of the *env* gene in a cohort of 23 HIV controllers from Brazil, divided in three groups, according to the level of systemic viral suppression: (1) elite controllers with persistent undetectable viral load (PEC, *n* = 6); (2) elite controllers with occasional episodes of transient (51–400 copies/mL) viremia (EEC, *n* = 7); and (3) viremic controllers with persistent low-level (80–2000 copies/mL) viremia (VC, *n* = 10).

**Results:**

The HIV-1 diversity of the PBMC-associated proviral quasispecies in EC was significantly (*P* < 0.01) lower than in VC, but not significantly different between PEC and EEC groups. We detected a considerable variation in the average pairwise nucleotide distance and proportion of unique sequences in the HIV-1 proviral quasispecies of PEC and EEC. Some PEC and EEC displayed highly homogenous proviral populations with large clusters of identical sequences, while others exhibited relatively diverse proviral populations with a high proportion of unique sequences comparable to VC subjects. The long-term (10–15 years) follow-up of the HIV-1 proviral populations revealed a complete evolutionary stasis in one PEC and measurable divergence rates in one EEC [3.1 (1.2–5.6) × 10^−3^ substitutions/site/year and one VC [2.9 (0.7–5.1) × 10^−3^ substitutions/site/year].

**Conclusions:**

There is no simple relationship between systemic viral suppression and intra-host proviral diversity or rate of reservoir’s reseeding in chronically infected HIV controllers. Our results demonstrate that very divergent patterns of intra-host viral diversity and divergence could be detected in the setting of natural suppression of HIV-1 replication and that ongoing evolution and reseeding of the PBMC proviral reservoir occurs in some elite controllers.

**Electronic supplementary material:**

The online version of this article (doi:10.1186/s12977-017-0354-5) contains supplementary material, which is available to authorized users.

## Background

The natural history of human immunodeficiency virus type-1 (HIV-1) infections may display very divergent patterns among individuals. Most HIV-1 infected individuals, termed typical progressors (TP), display high plasma viral loads and progress to AIDS without treatment after 5–10 years of infection [1]. Some individuals, termed long-term non-progressors (LTNPs), display longer asymptomatic periods (>10 years) and keep normal CD4^+^ T cell counts in the absence of treatment [1]; while others, termed HIV controllers, exhibit a durable control of viral replication maintaining at very low levels during chronic infection [2]. Among HIV controllers, the viremic controllers (VC) suppress the viremia to levels <2000 HIV-1 RNA copies/mL and the elite controllers (EC) to levels <50-80 HIV-1 RNA copies/mL.

Intra-host HIV-1 evolution in TP follows a consistent pattern of temporal changes in viral diversity and divergence during the course of infection, that affect both proviral DNA populations in peripheral blood mononuclear cells (PBMC) and viral RNA populations in plasma [3]. According to that pattern, infection is usually initiated by a relatively homogeneous viral population (with less than 1% envelope [*env*] diversity) that diversifies during the asymptomatic phase, reaching a peak of population diversity (up to 10% at the *env* gene) and divergence before leveling off or decrease towards the AIDS phase. A roughly similar pattern of intra-host HIV-1 evolution was described for LTNPs and HIV controllers in the plasma compartment [3]. LTNPs display HIV-1 RNA populations that continuously evolve during chronic infection and reach an overall diversity comparable to that observed in TP [4]. Several studies also demonstrate ongoing evolution and divergence of HIV-1 RNA sequences from most EC [5–12], although the mean diversity of plasma populations in EC is significantly lower than that observed for TP at chronic infection [8].

The HIV-1 diversity and divergence pattern of PBMC-associated proviral sequences from LTNPs and HIV controllers, however, differed strikingly from that observed in the plasma virus. In some LTNPs, DNA proviral populations are composed of a complex mixture of archival (dating close to the patient’s seroconversion time) and recent (dating close to the sampling time) variants [13] and displayed no temporal structure in the changes of diversity and divergence during chronic infection [14]. In all chronically infected EC and some VC, DNA proviral populations are extremely homogenous (with less than 2% *env* diversity), mostly composed by ancestral sequences and with no measurable divergence over time [5, 9, 10, 12, 15–19]. A recent study demonstrates that most proviral sequences detected in PBMC from HIV controllers are largely representative of archival variants probably integrated during primary infection and propagated by clonal expansion of the memory CD4^+^ T cell latent reservoir, although rare proviral clones of recent origin could be detected in some patients [12].

These observations suggest that the virus is evolving in HIV controllers, but most evolving plasma viruses do not replenish the PBMC reservoir and the majority of PBMC-associated proviral sequences detected in chronically infected HIV controllers represent ancestral variants. The precise correlation between the systemic viral suppression level and the rate of reservoir’s reseeding in HIV controllers, however, is not fully understood. Furthermore, previous studies may have failed to detect proviral sequence replenishment and ongoing evolution in HIV controllers because of the narrow follow-up time (usually 2–6 years). To answer these questions, we performed a cross-sectional analysis of the DNA proviral quasispecies diversity at the *env* gene in 23 HIV controllers with different levels of systemic viral suppression and we also recover the long-term (10–15 years) pattern of changes of HIV-1 proviral populations in the setting of low/undetectable viremia.

## Methods

### Study subjects

A cohort of 23 HIV controllers, defined as subjects infected with HIV-1 for at least 5 years and maintaining RNA viral loads of <2000 copies/mL without antiretroviral therapy, has been followed-up at the Instituto Nacional de Infectologia Evandro Chagas (INI), Rio de Janeiro, Brazil. These subjects were classified in two categories according to the plasmatic viral load (VL) during follow-up [20]: (1) elite controllers (EC) if most (≥70%) plasma VL determinations were below the limit of detection for the respective available assay (<50–80 copies/mL) (n = 13) and (2) viremic controllers (VC) if most (≥70%) VL determinations were between 80 and 2000 copies/mL (n = 10). The EC were further subdivided in two subgroups [21]: persistent elite controllers (PEC) if 100% of VL measures were below the limit of detection (n = 6) and (2) ebbing elite controllers (EEC) if subjects had occasional (<30% of frequency) episodes of transient low-level (51–400 copies/mL) viremia (n = 7). Patients were followed at least once every 6–12 months to perform infection-monitoring tests such as RNA viral load quantification and CD4^+^ T lymphocyte count. In each visit, PBMC were obtained by Histopaque-1077 (Sigma, USA) density gradient and stored in liquid nitrogen until use. The present work was approved by the Brazilian National Human Research Ethics Committee (CONEP 14430/2011) and all subjects gave written informed consent.

### CD4^+^ T cell counts and plasma HIV-1 RNA quantification

Absolute CD4^+^ T cell counts were obtained using the MultiTest TruCount-kit and the MultiSet software on a FACSCalibur flow cytometer (BD Biosciences San Jose, CA). Plasma VL were measured according to the Brazilian Ministry of Health guidelines, with methodologies being updated overtime to improve sensitivity: Nuclisens HIV-1 RNA QT assay (Organon Teknika, Durham, NC, limit of detection: 80 copies/mL) from 1999 to 2007; the Versant HIV-1 3.0 RNA assay (bDNA 3.0, Siemens, Tarrytown, NY, limit of detection: 50 copies/mL) from 2007 to 2013; and the Abbott RealTime HIV-1 assay (Abbott Laboratories, Wiesbaden, Germany, limit of detection: 40 copies/mL) from 2013 to 2016.

### HIV-1 DNA extraction and single genome sequencing

Cryopreserved PBMC were thawed, washed and immediately after, the total genomic DNA was isolated with addition of the DNAzol^®^ Reagent (Invitrogen, USA) as described [22]. To limit template resampling, single genome amplification (SGA) was performed by limiting dilution nested PCR at a concentration of DNA that would produce less than 40% of positive PCR reactions, providing a >70% probability that a positive PCR originates from a single molecule [23]. A fragment of nearly 600 bp of the HIV-1 *env* gene (including the C2–C4 regions of gp120) was amplified by PCR using AmpliTaq Gold^®^ 360 DNA Polymerase (Applied Biosystems, USA) as described [17]. The final PCR products were purified using the Illustra GFX PCR DNA purification kit (GE Healthcare, USA) and directly sequenced using the ABI BigDye Terminator v.3.1 reaction Kit (Applied Biosystems, Foster City, CA) in an ABI PRISM 3100 automate sequencer (Applied Biosystem). Chromatograms were assembled into contigs using the SeqMan 7.0 software (DNASTAR Inc., Madison, WI). Sequences resulting from low-quality chromatograms, from chromatograms with double peaks (indicative of more than one template per sequencing reaction), or showing APOBEC3G/F-mediated hypermutation as determined using Hypermut software [24] were discarded.

### HIV-1 subtyping

*Env* sequences from HIV controllers were aligned with HIV-1 subtype reference sequences using ClustalW and then manually edited, yielding a final alignment covering positions 7008–7650 relative to the HXB2 reference genome. Maximum-likelihood (ML) phylogenetic trees were reconstructed with the PhyML 3.0 program [25] using the most appropriate nucleotide substitution model selected using program jModeltest v. 3.7 [26], the SPR branch swapping heuristic tree search algorithm, and the approximate likelihood-ratio test (aLRT) [27] for branch support.

### Prediction of coreceptor usage and CCR5 genotyping

The V3 region of *env* sequences was translated using MEGA7 [28] and viral tropism was predicted using Geno2pheno (http://coreceptor.bioinf.mpisb.mpg.de/cgi-bin/coreceptor.pl) with a false positive rate (FPR) cutoff of 5% [29]. The presence of the ∆32 variant in CCR5 was assessed by PCR amplification/agarose gel electrophoresis as previously described [21].

### Analyses of viral diversity and divergence

The complexity of proviral quasispecies was characterized using two indices: the mean nucleotide diversity (π) and the normalized Shannon entropy (*H*_SN_). The π measures the average number of nucleotide differences between any two sequences of the quasispecies obtained at the same time point and was calculated using MEGA7 [28] as described previously [17]. The *H*_SN_ provides a measure of haplotype (mutant) frequencies and was calculated by using the R package, Vegan [30], after rarefaction of samples to the small sample size (*n* = 10) for bias correction of sample size differences [31]. The divergence rate of proviral *env* sequences was estimated for three patients (one PEC, one EEC and one VC) with available sequences sampled between 5 and 15 years ago [17]. ML phylogenetic trees were reconstructed for each patient as described above and linear regression analysis of the root-to-tip distances against sampling time were performed using program Tempest [32] to verify the temporal structure of the datasets. The intra-host viral evolutionary (divergence) rate was then directly estimated from the sampling date of the sequences for those datasets with a good temporal structure using program BEAST v1.8 [33]. Analyses were performed using the most appropriate nucleotide substitution model for each patient, a relaxed uncorrelated lognormal molecular clock model [34] with a CTMC rate reference prior [35] and a Bayesian coalescent tree prior [36]. Three MCMC chains were run for 1 × 10^7^ generations and then combined. Effective Sample Size (ESS) and 95% Highest Probability Density (HPD) values were inspected using Tracer v1.6 (http://tree.bio.ed.ac.uk/software/tracer/) to assess the convergence and uncertainty of parameter estimates.

### Statistical analysis

Statistical analyses were performed using GraphPad v6 (Prism Software, USA). The Mann–Whitney test was use to compare the quasispecies diversity, the time since HIV-1 diagnosis and the CD4^+^ T cell counts between subjects groups. Tests were considered significant if the *P* value was ≤0.05.

## Results

### Epidemiological, clinical and virological characteristics of HIV controllers

**Fig. 1:**
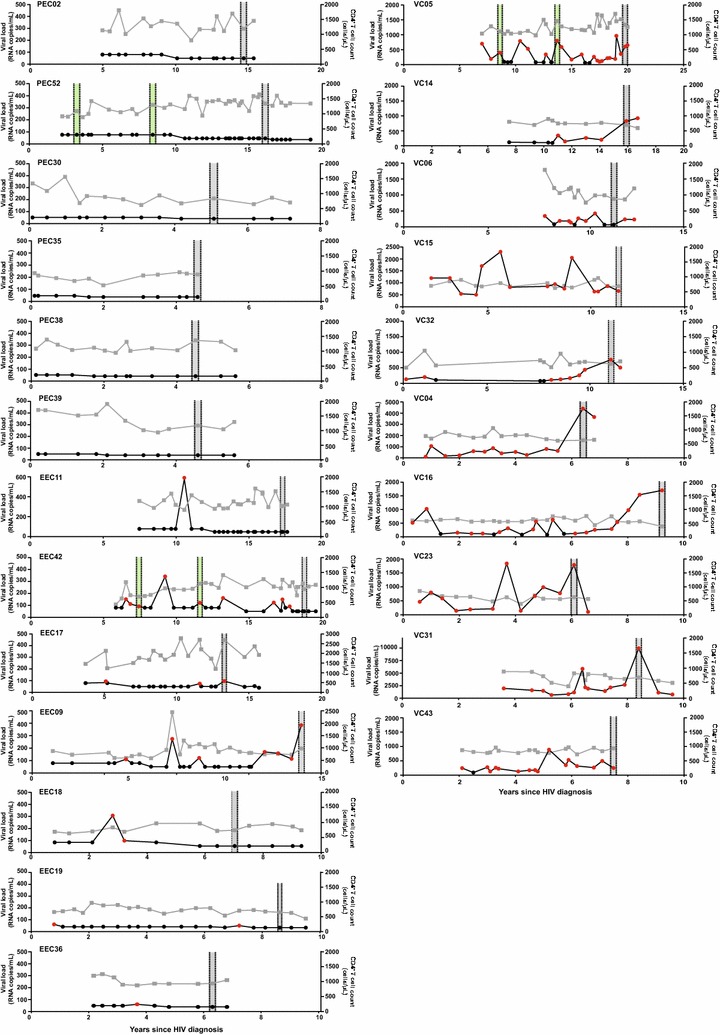
Clinical follow-up of the 23 HIV-1 controllers. Plasma RNA viral load (copies/mL, *circles*) and CD4^+^ T cell counts (cells/µL, *squares*) values over time (years) are shown on the *left* and *right Y* axis respectively. RNA viral loads below or above the detection limit are colored *black* and *red*, respectively. The limit of detection of RNA viral load varied over time according to the methodology used. *Shaded areas* indicate the time points selected in this study (*gray*) and previously (*green*) [17] for the DNA quasispecies analysis. Patient identification is shown in the *upper left corner* of each graph

The main clinical and epidemiological characteristics of our HIV controllers’ cohort are shown in Table 1. Female gender (61%) was more frequent than male (39%), 70% of the patients identified themselves as heterosexual and 22% as men who have sex with men (MSM), while information regarding exposure behavior was not available for 9%. A higher proportion of females (77 vs 40%) and heterosexuals (69 vs 40%) was observed in the EC group than in the VC group. Participants had a median age of 49 years (IQR: 41–53 years old) and had documented HIV infection for a median of 11 years (IQR: 6–15 years). The EC and VC groups have a similar median age (52 vs 46 years, respectively) and median documented time of HIV infection (9 vs 10 years, respectively) at sampling time (Additional file 1: Figure S1A, B). None of the HIV controllers exhibited AIDS-related conditions and the CD4^+^ T cell counts were ≥500 cells/µL during follow-up (Fig. 1). Most of them (83%) also had documented HIV-infection for over 8 years, thus being classified as LTNPs. EC, however, displayed a higher median CD4^+^ T-cell count than VC at sampling time (1202 vs 735 cells/μL, respectively) (Additional file 1: Figure S1C). No significant differences in clinical and epidemiological characteristics were observed between PEC and EEC subgroups (data not shown).

**Table 1:** Clinical and epidemiological characteristics of HIV controllers

Patient	Birth date (year)	Gender	Exposure category	Last HIV negative test (year)	First HIV positive test (year)	Median HIV RNA VL (range)	Median CD4^+^ T cells (IQR)
PEC02	1963	Female	HET	ND	1997	<LD	1272 (1128–1425)
PEC52	1971	Female	HET	ND	1997	<LD	1391 (1343–1461)
PEC30	1983	Male	HET	ND	2009	<LD	842 (669–968)
PEC35	1980	Female	HET	2004	2011	<LD	859 (767–943)
PEC38	1976	Female	Unknown	ND	2011	<LD	1080 (1020–1230)
PEC39	1944	Female	Unknown	ND	2011	<LD	1411 (1072–1640)
EEC09	1969	Male	MSM	ND	2001	<LD (<LD–388)	932 (807–1069)
EEC11	1967	Female	HET	1989	1995	<LD (<LD–580)	1127 (1007–1301)
EEC42	1954	Female	HET	1992	1993	<LD (<LD–341)	991 (924–1118)
EEC17	1950	Female	HET	ND	2000	<LD (<LD–96)	1874 (1674–2132)
EEC18	1933	Female	HET	ND	2001	<LD (<LD–300)	694 (667–809)
EEC19	1968	Male	HET	ND	2006	<LD (<LD–73)	889 (820–973)
EEC36	1976	Female	HET	2005	2010	<LD (<LD–61)	945 (937–1157)
VC04	1965	Female	HET	ND	2008	557 (108–4407)	779 (689–811)
VC05	1964	Male	HET	ND	1991	241 (55–800)	1254 (1101–1410)
VC06	1978	Male	MSM	1999	2000	169 (<LD–405)	1093 (960–1215)
VC14	1970	Female	HET	1996	1999	106 (55–782)	702 (688–757)
VC15	1974	Female	HET	ND	2001	855 (510–2052)	699 (681–825)
VC16	1967	Male	MSM	ND	1998	240 (<LD–1683)	556 (532–608)
VC23	1971	Male	HET	2004	2008	628 (139–1842)	635 (569–671)
VC31	1963	Male	MSM	2006	2006	1558 (587–10,026)	733 (654–814)
VC32	1978	Male	MSM	2004	2005	153 (<LD–722)	641 (564–709)
VC43	1973	Female	HET	ND	2008	232 (66–864)	850 (775–911)

*MSM* men who have sex with men, *ND* not determined, *VL* viral load (copies/mL), *LD* limit of detection, *IQR* interquartile range, median CD4^+^ T cell (cells/µL)

**Fig. 2:**
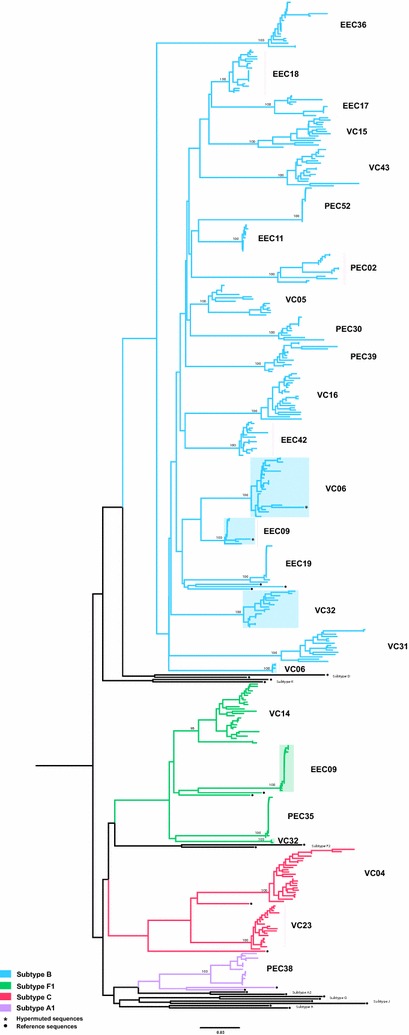
ML phylogenetic tree of *env* sequences from HIV-1 controllers and HIV-1 subtype reference sequences. Branches were colored according to the subtype assignment as shown in the legend at *bottom left*. The individual’s identification is displayed on the right side of the clusters. Sequence clusters from dual infected individuals (EEC09, VC06 and VC32) are indicated by *shaded boxes*. Bootstrap support for each individual cluster is shown. *Black circles* point to the reference sequences and *asterisks* highlight the sequences with APOBEC3G-mediated G to A hypermutations. Horizontal branch lengths are proportional to the *bar at the bottom* indicating nucleotide substitutions per site

A cross-sectional analysis of the HIV-1 proviral quasispecies in the 23 HIV controllers was performed by SGA of the *env* gene at between 5 and 20 years after HIV-diagnosis (Fig. 1). Similar median numbers of *env* clones per sample were obtained in EC (16, IQR: 14–18) and VC (18, IQR: 14–21) groups, as well as in PEC (15; IQR: 14–16) and EEC (17; IQR: 15–21) subgroups (Table 2). ML phylogenetic analysis revealed that *env* sequences from most individuals (*n* = 20, 87%) clustered by subject in highly supported (bootstrap >95%) monophyletic lineages (Fig. 2), thus supporting infection by a single variant. For three individuals (EEC09, VC06 and VC32), however, the *env* sequences branched in two independent monophyletic clades (Fig. 2), indicating dual infection. The subject VC06 was double infected by two HIV-1 subtype B variants, while individual EEC09 and VC32 were double infected by HIV-1 subtypes B and F1 variants. A second sample from these three individuals was analyzed confirming the previous result (data not shown). Overall, subtype B (74%) was the most frequent HIV-1 variant detected in our cohort, followed by subtypes F1 (13%), C (9%), and A1 (4%) (Fig. 2; Table 2). Prediction of coreceptor usage showed that most individuals (78%) presented only R5-tropic viral clones, two individuals (one EEC and one VC) presented a low frequency (5%) of X4-tropic clones, two individuals (one PEC and one VC) displayed a high frequency (30–45%) of X4-tropic viruses, and one PEC had only X4-tropic viral clones (Table 2). None of the subjects with high frequency of X4-tropic viruses is homozygous/heterozygous for the CCR5/Δ32 genotype (Table 2). Hypermutated proviral sequences were detected at a very low frequency (<5%) in only two individuals (Fig. 2).

**Table 2:** Virological characteristics of HIV controllers

Patient	Number of *env* clones	Subtype	HIV-1 tropism^a^		π (%)	H_SN_	Proportion of unique sequences (%)
PEC02	14	B	100% R5		4.4	0.72	57
PEC52	16	B	100 X4		0.1	0.36	18
PEC30	11	B	55% R5	45% X4	2.9	0.88	81
PEC35	19	F1	100% R5		0.1	0.23	21
PEC38	15	A	100% R5		3.9	0.80	60
PEC39^a^	14	B	100% R5		4.2	0.97	93
EEC09	12	B	100% R5		0.5	–	45
	21	F1	100% R5		0.2	0.59	38
EEC11^a^	13	B	100% R5		0.2	0.57	38
EEC42	16	B	100% R5		1.9	1.00	100
EEC17	10	B	100% R5		4.6	0.94	90
EEC18	20	B	95% R5	5% X4	2.7	0.87	75
EEC19	17	B	100% R5		0.6	0.26	23
EEC36	21	B	100% R5		1.9	0.82	62
VC04	24	C	100% R5		4.8	1.00	100
VC05	14	B	100% R5		5.9	0.93	86
VC06^a^	24	B	100% R5		2.1	0.92	70
	5	B	100% R5		0.02	–	60
VC14	12	F1	100% R5		4.4	0.99	96
VC15	14	B	100% R5		4.4	1.00	100
VC16	21	B	95% R5	5% X4	4.4	1.00	100
VC23	20	C	100% R5		2.5	1.00	100
VC31^a^	15	B	100% R5		6.4	0.98	93
VC32	17	B	100% R5		3.5	0.98	94
	2	F1	100% R5		0.2	–	100
VC43	17	B	70% R5	30% X4	4.1	0.96	88

^a^Heterozygous patients for Δ32 CCR5 allele

### Diversity of proviral quasispecies in HIV controllers

**Fig. 3:**
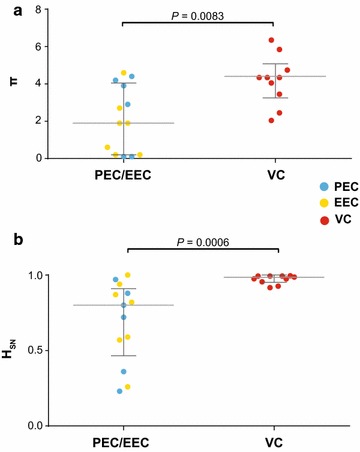
Mean nucleotide diversity (π, **a**) and normalized Shannon entropy (*H*_SN_, **b**) of proviral *env* quasispecies from EC and VC. The colors of the *circles* represent the different levels of systemic viral suppression in HIV-1 controllers as indicated in the legend. *Dotted* and continuous *gray lines* represent the median and interquartile ranges, respectively. *P* values were calculated using the Mann–Whitney test

To address the potential relationship between systemic viral suppression level and reservoir’s reseeding among the 23 HIV controllers of our cohort, we calculated π and *H*_SN_ indices that measure the average pairwise nucleotide distance and the mutant frequencies (proportion of unique sequences) in the set of aligned sequences of each individual, respectively. For double-infected patients, only sequences of the prevalent HIV-1 variant were considered. VC displayed quite diverse (π > 2%) and complex (*H*_SN_ > 0.90) proviral quasipecies that were mostly (>70%) composed by unique sequences (Table 2; Additional file 2: Figure S2). The overall mean π and *H*_SN_ estimated for HIV-1 quasispecies in the VC group were significantly higher than those estimated for the EC group (*P* < 0.01), despite the fact that the time since HIV-diagnosis was comparable among groups (Fig. 3). This supports that the PBMC reservoir of VC display higher rate of evolution and reseeding than that of EC.

**Fig. 4:**
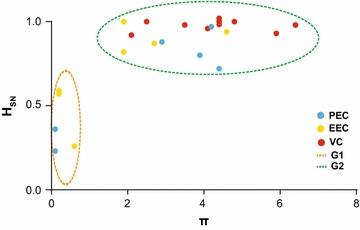
Identification of divergent patterns of intra-host viral diversity among EC subjects. The normalized Shannon entropy (*H*_SN_ *, y* axis) of each subject’s proviral *env* quasispecies was plotted against the corresponding mean nucleotide diversity (π, *x* axis). The colors of the *circles* represent the different levels of systemic viral suppression in HIV-1 controllers as indicated in the legend. *Dashed circles* identify the two main patterns of intra-host viral diversity: proviral quasispecies of individuals from group 1 (G1, *dashed orange line*) displayed low diversity (π < 1%) and a high proportion of identical sequences (*H*_SN_ < 0.6); proviral quasispecies of individuals from group 2 (G2, *dashed green line*) exhibited larger diversity (π ≥ 2%) and a higher frequency of unique sequences (*H*_SN_ > 0.7)

A closer inspection of the EC group, however, reveals that both diversity and complexity of HIV-1 quasispecies extensively varied among subjects (Table 2; Fig. 3). The combined analysis of π and *H*_SN_ allow us to detect two divergent patterns of intra-host viral diversity within the EC group (Fig. 4; Additional file 2: Figure S2). The first group (G1) comprises five EC (two PEC and three EEC) that present highly homogenous (π < 1%) proviral quasispecies with large clusters of identical sequences (*H*_SN_ < 0.6). The second group (G2) comprises eight EC (four PEC and four EEC) showing relatively diverse (π ≥ 2%) proviral populations with high proportion of unique sequences (*H*_SN_ > 0.7), comparable to those observed among VC subjects. Similar median values of π and *H*_SN_ were estimated for EC with or without detection of occasional viremia above the limit of detection (Additional file 3: Figure S3). Quasispecies diversity and complexity were also not correlated with time since HIV diagnosis in EC (Additional file 4: Figure S4).

### Rates of evolution of proviral quasispecies in HIV controllers

**Fig. 5:**
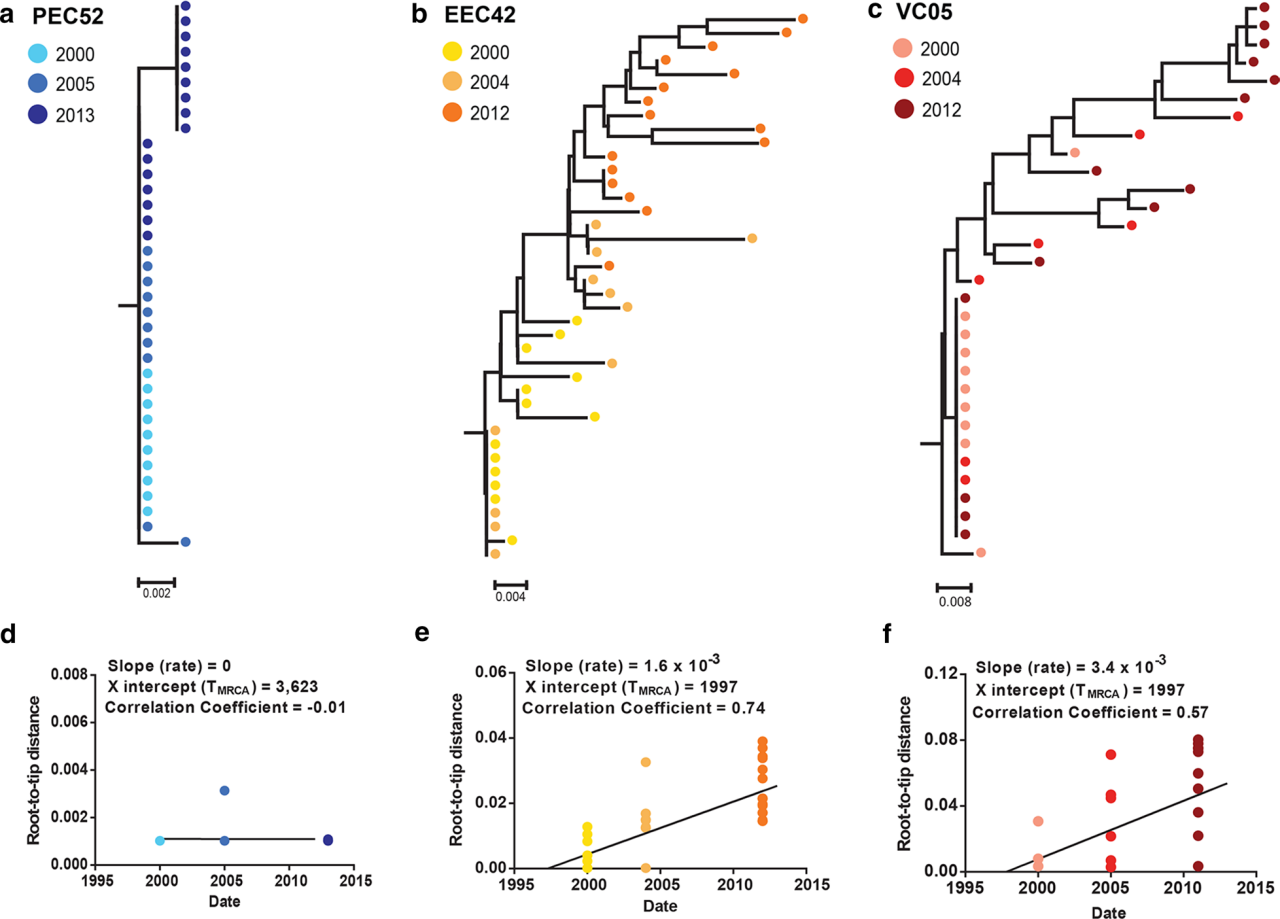
Longitudinal analysis of HIV-1 proviral *env* sequences obtained from subjects PEC52 (**a**, **d**), EEC42 (**b**, **e**) and VC05 (**c**, **f**) between 2000–2013, 2000–2012 and 2000–2011, respectively. **a**–**c** ML phylogenetic trees for each individual are shown, in which horizontal branch lengths are drawn to scale with the *bar at the bottom* indicating nucleotide substitutions per site. **d**–**f** Plots of the root-to-tip distance against sequence sampling time are shown below each subject tree. The slope, coefficient of regression and X intercept of linear regression analysis is indicated. The colors of the *circles* in phylogenetic trees and plots represent the sampling year at which sequences were obtained

The pattern of intra-host viral diversity observed in EC-G1 is consistent with amplification of viral reservoir mostly by clonal expansion of infected memory CD4^+^ T cells; whereas the pattern observed in EC-G2 and VC patients supports a continuous reseeding of the proviral reservoir. To confirm that hypothesis, we investigated the long-term evolution of the PBMC proviral compartment in three individuals from groups EC-G1 (PEC52), EC-G2 (EEC42) and VC (VC05) by combining the *env* proviral sequences obtained in the present study with those obtained from the same patients 10–13 years ago and that were described previously [17]. ML phylogenetic trees were reconstructed for each patient and the root-to-tip distances were plotted against sampling time. Despite the very long follow-up time (13 years), proviral *env* sequences of patient PEC52 were mostly identical and with no evidence of increasing root-to-tip distance over time (Fig. 5), thus confirming absence of reseeding and evolution of the PBMC reservoir in this patient. All proviral *env* sequences from patient EEC42 and most (70%) *env* sequences from patient VC05 sampled at the most recent time-point, by contrast, were different from those sampled 11–12 years earlier and with clear evidence of evolution (increasing root-to-tip distance over time) (Fig. 5). To estimate the intra-host HIV-1 evolutionary rate in subjects EEC42 and VC05, *env* sequences from different time points were analyzed using the BEAST program. For each cluster of identical sequences, only those *env* sequences sampled at the earliest time point were retained to reduce the impact of latency on intra-host evolutionary rate estimations. According to these analyses, the mean intra-host evolutionary rate of proviral *env* sequences estimated for patient EEC42 was 3.1 × 10^−3^ subst/site/year (95% HPD: 1.2–5.6 × 10^−3^ subst/site/year) and for patient VC was 2.9 × 10^−3^ subst/site/year (95% HPD: 0.7–5.1 × 10^−3^ subst/site/year).

## Discussion

In this study, we examined the DNA proviral quasispecies diversity at the *env* gene in 23 chronically infected HIV controllers with different levels of systemic viral suppression. Most HIV controllers included in our cohort were females (61%) and this percentage was higher for EC (77%) than for VC (40%). This may be a consequence of the greater frequency of women seeking health services for routine and preventive exams than men, enabling the diagnosis of HIV even in the absence of symptoms [37], and/or may reflect gender-specific differences in the plasma HIV-1 RNA levels [38–40]. Although no HIV controllers exhibited AIDS-related conditions and had CD4^+^ T cell counts ≥500 cells/µL during follow-up, EC (1202 cells/μL) displayed a significantly higher median CD4^+^ T cell counts than VC (735 cells/μL) at sampling time, supporting the relevance of persistent low-level viremia on the long-term CD4^+^ T cell decline [20, 41].

Analysis of proviral *env* sequences from HIV controllers revealed a diverse molecular epidemiologic profile with detection of HIV-1 subtypes B (74%), F1 (13%), C (9%) and A1 (4%). While subtypes B, F1 and C are common HIV-1 clades circulating in Brazil [42], subtype A1 has been only described in one case [43]. Three individuals (one EC and two VC) were dually infected with strains of the same (B) or different (B and F1) subtypes, resulting in a prevalence of dual HIV-1 infection (13%) comparable to that previously estimated in a Spanish cohort of LTNP-EC (20%) [44]. Prediction of coreceptor usage further revealed a significant frequency (30–100%) of X4-tropic clones in proviral quasispecies of two PEC and one VC. Reanalysis of proviral *env* sequences from EC and VC already published [12, 16, 18] showed that high frequency (>30%) of X4-tropic clones is a rare phenomenon, being detected in only one out of 25 subjects analyzed (data not shown). These results demonstrate that natural suppression of HIV-1 viremia below 2000 copies/mL can be achieved in the context of either single or dual HIV-1 infections, regardless of the subtype and coreceptor usage of infecting virus.

The HIV-1 proviral population continuously diversifies during untreated asymptomatic infection, although the rate of diversification greatly varies among individuals. In TP with RNA viral loads above 10,000 copies/mL, *env* gene diversity increases at a mean rate of 1%/year and reaches a peak (π = 6–10%) after 5–10 years post-infection [3]. High levels of *env* proviral diversity (π = 4-8%) have been also described in samples taken 10–15 years after HIV diagnosis from LTNPs with plasma viremia between 2000 and 10,000 copies/mL [4, 16, 17, 19, 45]. Much lower levels of *env* proviral diversity (π = 0.1–6%), by contrast, were detected here in samples taken between 5 and 20 years after HIV diagnosis from HIV controllers (RNA viral load lower than 2000 copies/mL). This is consistent with previous studies [15–19] and with the notion that no viral diversification is expected when the host immune response greatly reduces the HIV-1 replication limiting the selection of escape mutants [46].

A closer inspection of the quasispecies diversity in different HIV controller groups here studied, however, revealed a more complex scenario. Particularly, the mean *env* diversity of proviral quasispecies in EC subjects varied over a large range (0.1–4.6%) and two distinct patterns of intra-host viral diversity were observed in that group. While some EC subjects (EC-G1) displayed highly homogeneous proviral populations (π < 1%) mainly composed by large clusters of identical sequences (*H*_SN_ < 0.6), other EC subjects (EC-G2) showed more diverse (π ≥ 2%) proviral populations comprising high proportions of unique sequences (*H*_SN_ > 0.7), comparable to those observed in VC subjects. Thus, contrary to initial expectations, the presence of a highly homogenous PBMC-associated HIV-1 proviral population is not a common characteristic of all EC subjects and no linear correlation could be observed between proviral quasispecies diversity and systemic viral suppression in HIV controllers.

Analysis of the long-term evolution of proviral populations revealed that the distinct patterns of intra-host viral diversity observed in HIV controllers might reflect different driving forces for the maintenance of the viral reservoir. Proviral *env* sequences of individual PEC52 (EC-G1 group) taken over a period of 13 years were mostly identical and displayed no evidence of divergence over time, demonstrating that most PBMC-associated proviral sequences detected in this chronically infected HIV controller represent ancestral variants that persist for >10 years of infection. This pattern supports the notion that the proviral reservoir, in some EC subjects, is mostly maintained by the clonal expansion of CD4^+^ T lymphocytes. Those cells were probably latently infected at the initial stage of infection, culminating in the absence of evolution and the preservation of a highly homogenous proviral population, similar to those observed in the majority of acutely infected patients [47–54].

In sharp contrast to patient PEC52, proviral populations of subjects EEC42 (EC-G2 group) and VC05 (VC group) displayed an increasing divergence and a partial or complete replacement of sequence variants over time. Although the mean *env* intra-host divergence rate here estimated for HIV controllers (~3 × 10^−3^ subst/site/year) was much lower than that previously estimated for TP (~10 × 10^−3^ subst/site/year) [3], the pattern observed is fully consistent with a continuous reseeding of the PBMC proviral reservoir in those HIV controllers. While several studies already demonstrate ongoing evolution and divergence of HIV-1 RNA sequences from the plasma compartment in VC and EC [5–12], this is the first study to quantify the intra-host divergence rate of DNA proviral sequences in the setting of undetectable viremia. These observations demonstrate that the HIV-1 in VC and in some EC is not only evolving, but also that the PBMC reservoir is continuously being resseded at a low, but measurable, rate leading to the partial or complete substitution of ancestral variants over time.

The divergent patterns of genetic diversity and evolution of proviral populations from EC here observed may be due to: (1) different levels of systemic suppression, (2) diverse mechanisms of natural control of HIV-1 replication, and/or (3) differences in the transmitted virus populations. Although a previous study conducted by our group demonstrated that rare episodes of detectable viremia in EC are associated to higher levels of systemic immune activation and a stronger HIV-1 specific immune response [21], pointing to lower levels of systemic viral supression in EEC than in PEC, we found no significant difference in the quasispecies diversity between both EC subgroups. It is possible that EC-G1 subjects display more efficient control mechanisms, capable of limiting new rounds of infection, particularly in the lymph nodes, than those present in EC-G2 subjects. Finally, it is also possible that the high proviral diversity detected in some EC was not due to intra-host evolution, but was present since the beginning of infection. Indeed, it was demonstrated that a substantial fraction of subjects (20–30%) displayed heterogeneous (2–5% *env* diversity) proviral populations in PBMC before seroconversion, most likely resulting from transmission of multiple HIV-1 variants [47–54]. If differences observed arise from multiple underlying mechanisms, definition of homogenous EC subgroups could become increasing challenging as more subjects are characterized.

## Conclusions

These results reveal that very divergent patterns of intra-host viral diversity and divergence could be detected in the setting of natural suppression of HIV-1 replication, suggesting that HIV-1 may evolve differently in every patient. We found no simple relationship between systemic viral suppression and intra-host proviral diversity or rate of reservoir’s reseeding in chronically infected HIV controllers, although the influence of some potential confounding factors such as the transmission of multiple HIV-1 variants in some EC cannot be ruled out. Our study also demonstrates that ongoing evolution and reseeding of the PBMC proviral reservoir is possible in some EC. The long-term longitudinal follow-up of more EC patients will be important to elucidate the major driving forces of the different intra-host evolutionary patterns here detected as well as their impact on the long-lasting control of HIV-1 replication and disease progression.

## Additional files

**Additional file 1: Figure S1.** Median of the Age (A), time since HIV diagnosis (B) and CD4+ T cell count (C) of HIV-1 controllers at the sampling point. The colors of the circles represent the different levels of systemic viral suppression in HIV-1 controllers as indicated in the legend. Dotted and continuous gray lines represent the median and interquartile ranges, respectively. *P* values were calculated using the Mann–Whitney test.

**Additional file 2: Figure S2.** ML phylogenetic trees of HIV-1 proviral *env* sequences obtained by SGA from PBMC of EC-G1, EC-G2 and VC subjects. Each tree represents the sequences from an individual. Presence of black and white circles in subjects EEC09, VC06 and VC32 is indicative of dual infection. Trees were rooted at the midpoint. Horizontal branch lengths are drawn to scale with the bar at the bottom indicating nucleotide substitutions per site. Sequences with G-to-A hypermutations were removed from this analysis.

**Additional file 3: Figure S3.** Mean nucleotide diversity (π, A) and normalized Shannon entropy (*H*_SN_) (B) of proviral *env* quasispecies from PEC and EEC. The colors of the circles represent the different levels of systemic viral suppression in HIV-1 controllers as indicated in the legend. Dotted and continuous gray lines represent the median and interquartile ranges, respectively. *P* values were calculated using the Mann–Whitney test.

**Additional file 4: Figure S4.** Mean nucleotide diversity (π, A) and normalized Shannon entropy (*H*_SN_, B) of proviral *env* quasispecies from PEC and EEC plotted against time since HIV diagnosis. The *P* value of linear regression analysis is indicated in each plot. The colors of the circles represent the subject classification according to the pattern of intra-host viral diversity (G1 and G2) described in Figure 4, as indicated in the legend at the right.
