# Supplementary figures and images for: Highly divergent patterns of genetic diversity and evolution in proviral quasispecies from HIV controllers
Source: Retrovirology. 2017 May 2;14:29. doi: 10.1186/s12977-017-0354-5 (PMC5414336; doi:10.1186/s12977-017-0354-5)

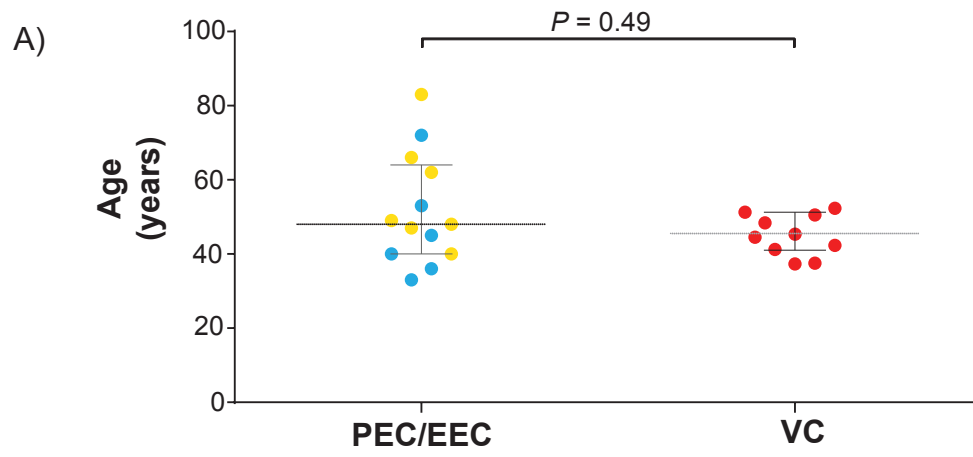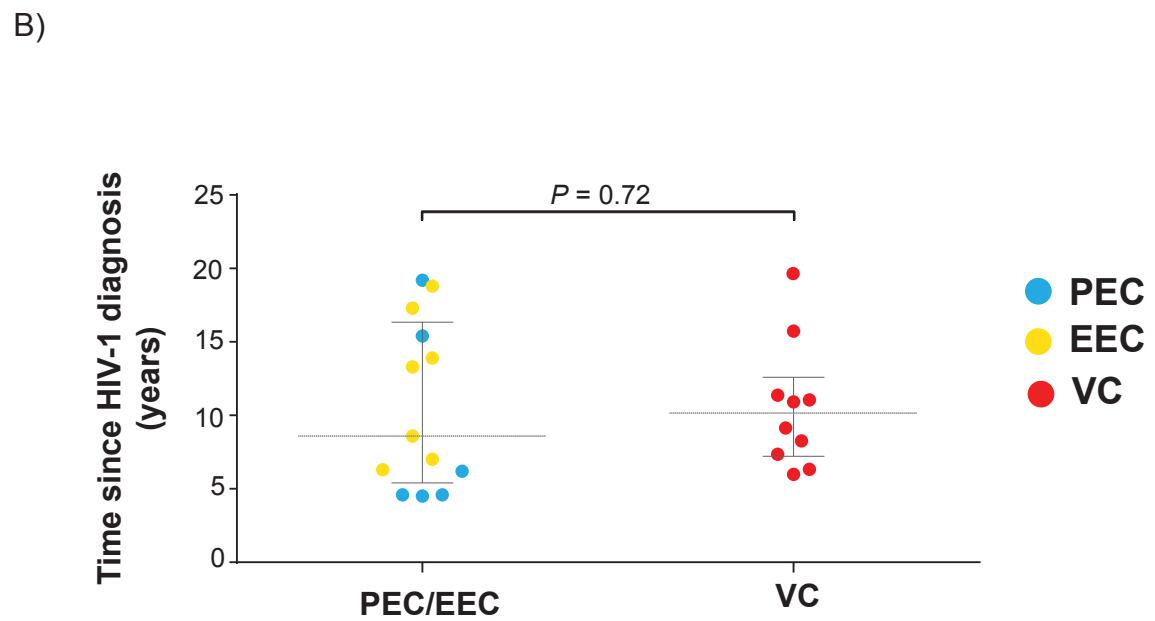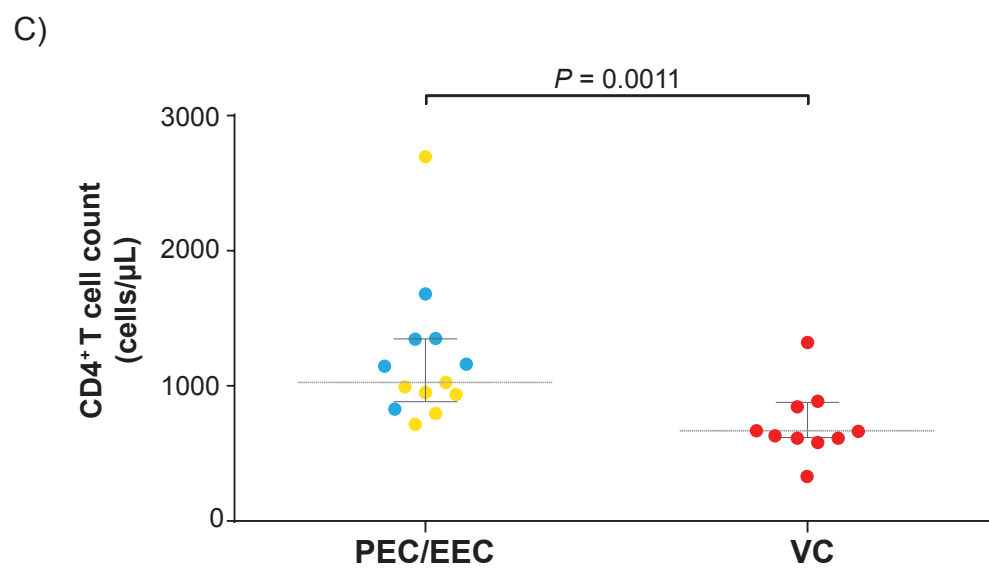

Supplement: Supplementary file 1 — Additional file 1: Figure S1. Median of the Age (A), time since HIV diagnosis (B) and CD4+ T cell count (C) of HIV-1 controllers at the sampling point. The colors of the circles represent the different levels of systemic viral suppression in HIV-1 controllers as indicated in the legend. Dotted and continuous gray lines represent the median and interquartile ranges, respectively. P values were calculated using the Mann–Whitney test. [file 12977_2017_354_MOESM1_ESM.pdf]

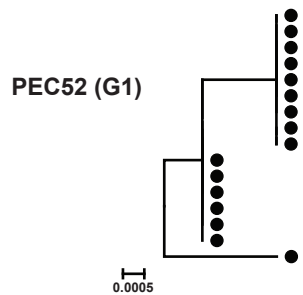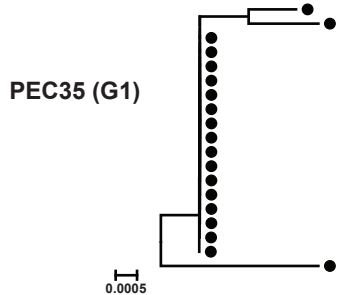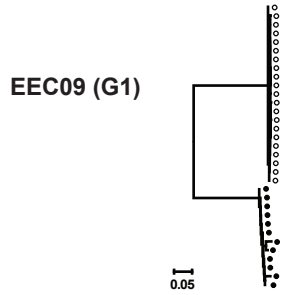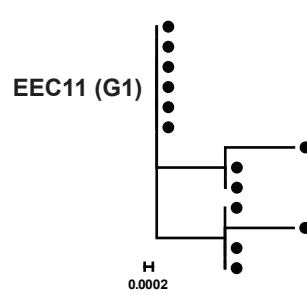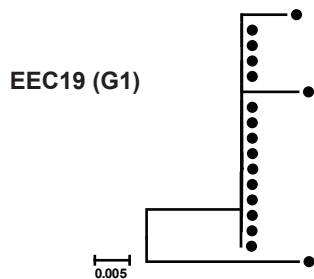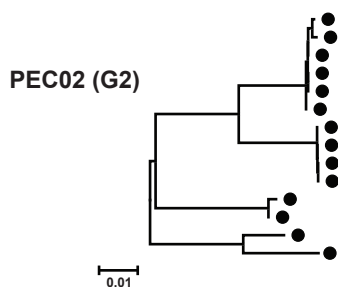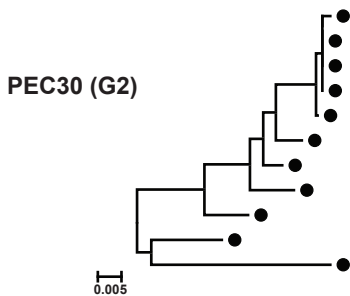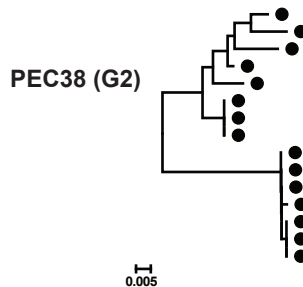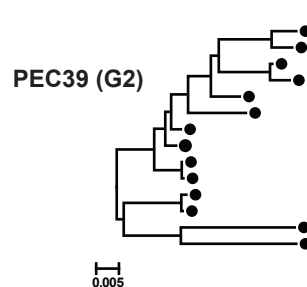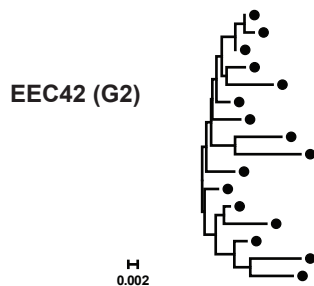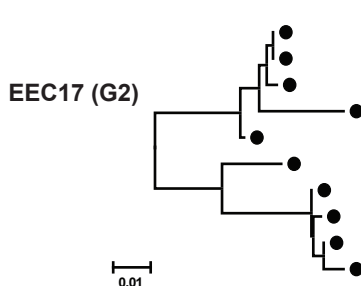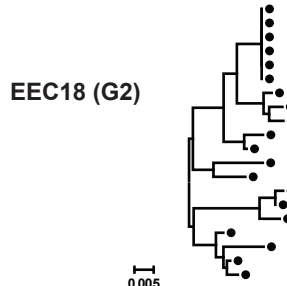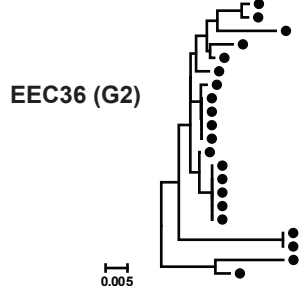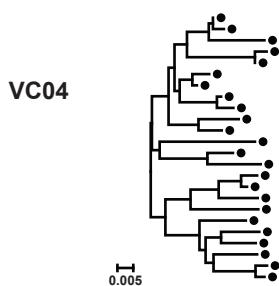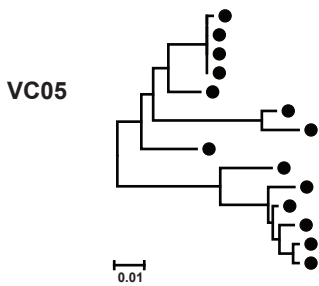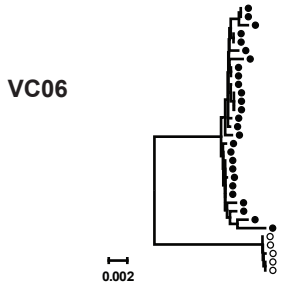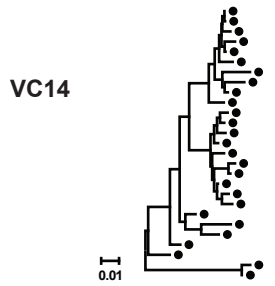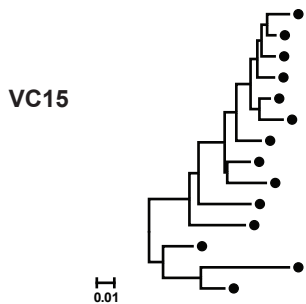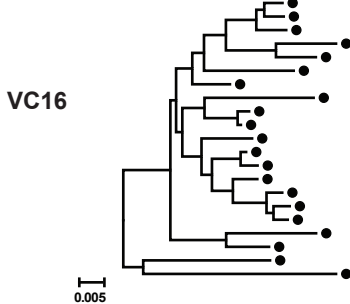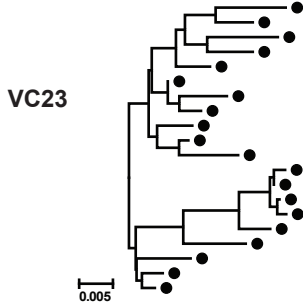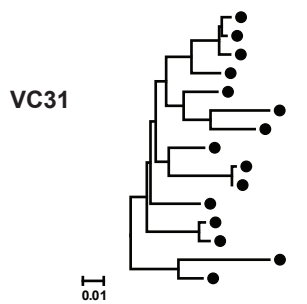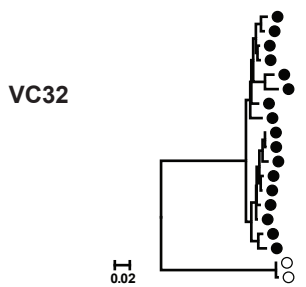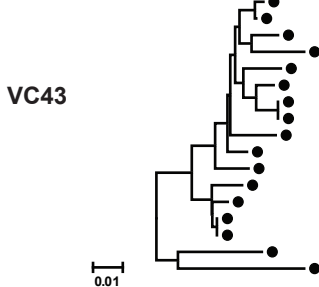

Supplement: Supplementary file 2 — Additional file 2: Figure S2. ML phylogenetic trees of HIV-1 proviral env sequences obtained by SGA from PBMC of EC-G1, EC-G2 and VC subjects. Each tree represents the sequences from an individual. Presence of black and white circles in subjects EEC09, VC06 and VC32 is indicative of dual infection. Trees were rooted at the midpoint. Horizontal branch lengths are drawn to scale with the bar at the bottom indicating nucleotide substitutions per site. Sequences with G-to-A hypermutations were removed from this analysis. [file 12977_2017_354_MOESM2_ESM.pdf]

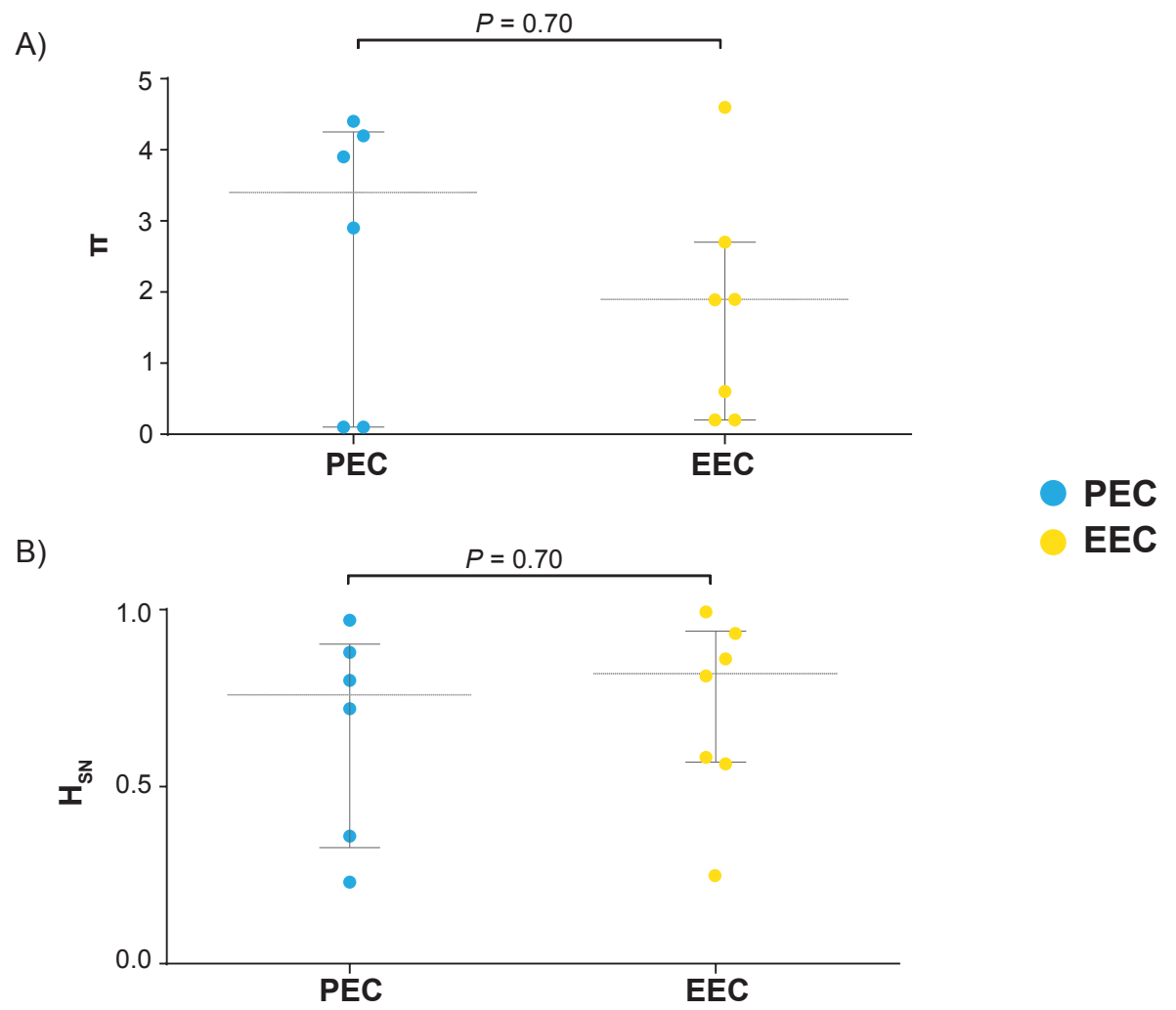

Supplement: Supplementary file 3 — Additional file 3: Figure S3. Mean nucleotide diversity (π, A) and normalized Shannon entropy (H SN) (B) of proviral env quasispecies from PEC and EEC. The colors of the circles represent the different levels of systemic viral suppression in HIV-1 controllers as indicated in the legend. Dotted and continuous gray lines represent the median and interquartile ranges, respectively. P values were calculated using the Mann–Whitney test. [file 12977_2017_354_MOESM3_ESM.pdf]

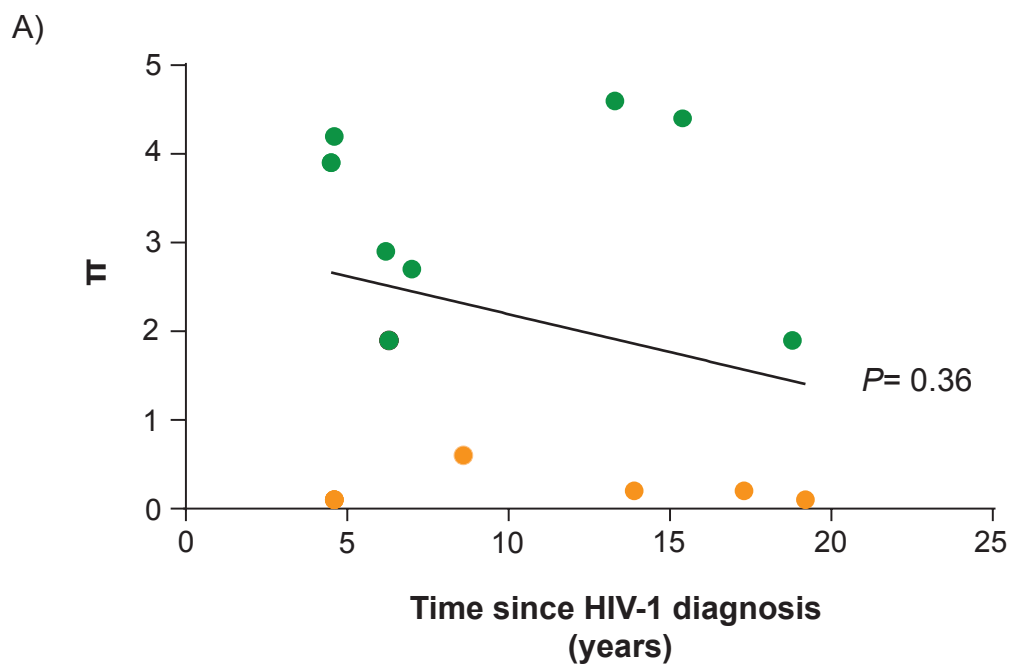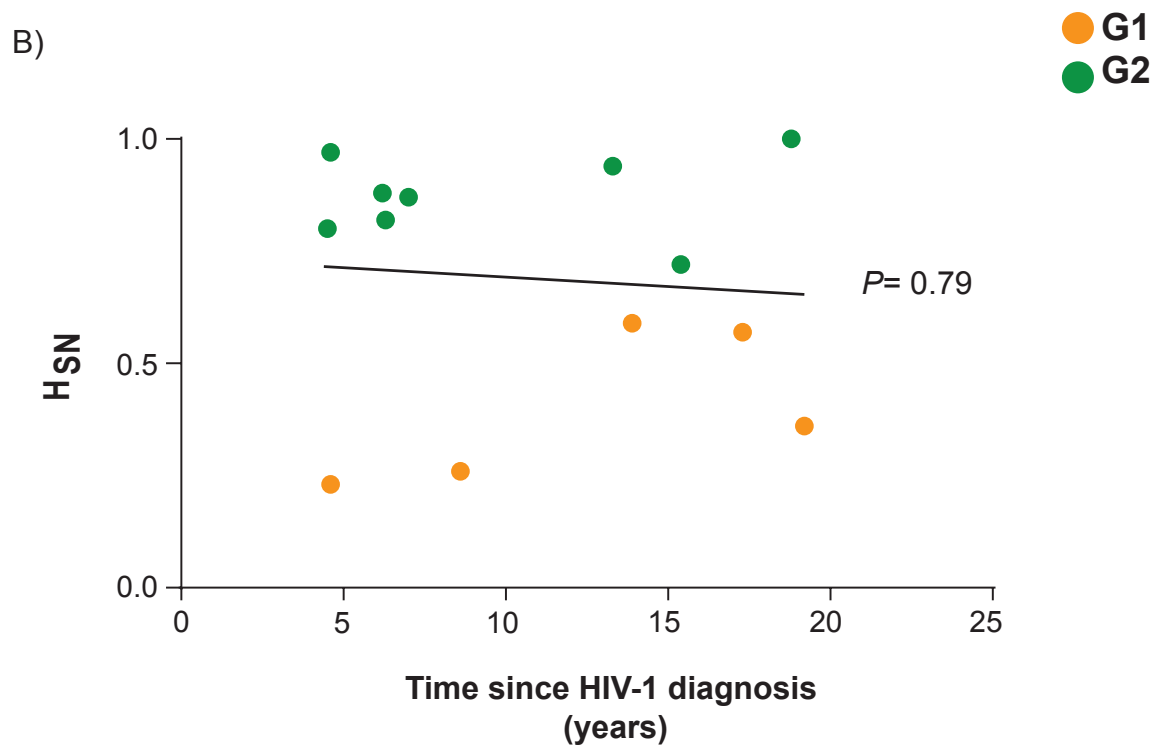

Supplement: Supplementary file 4 — Additional file 4: Figure S4. Mean nucleotide diversity (π, A) and normalized Shannon entropy (H SN, B) of proviral env quasispecies from PEC and EEC plotted against time since HIV diagnosis. The P value of linear regression analysis is indicated in each plot. The colors of the circles represent the subject classification according to the pattern of intra-host viral diversity (G1 and G2) described in Figure 4, as indicated in the legend at the right. [file 12977_2017_354_MOESM4_ESM.pdf]
